# GRIM-1, a Novel Growth Suppressor, Inhibits rRNA Maturation by Suppressing Small Nucleolar RNAs

**DOI:** 10.1371/journal.pone.0024082

**Published:** 2011-09-08

**Authors:** Shreeram C. Nallar, Limei Lin, Varsha Srivastava, Padmaja Gade, Edward R. Hofmann, Hafiz Ahmed, Sekhar P. Reddy, Dhananjaya V. Kalvakolanu

**Affiliations:** 1 Department of Microbiology and Immunology, University of Maryland School of Medicine, University of Maryland Marlene and Stewart Greenebaum Cancer Center, Baltimore, Maryland, United States of America; 2 Department of Biochemistry and Molecular Biology, University of Maryland School of Medicine, University of Maryland Marlene and Stewart Greenebaum Cancer Center, Baltimore, Maryland, United States of America; 3 Cancer Biology track Graduate Program in Life Sciences, University of Maryland School of Medicine, University of Maryland Marlene and Stewart Greenebaum Cancer Center, Baltimore, Maryland, United States of America; 4 Department of Pediatrics, University of Illinois at Chicago, Chicago, Illinois, United States of America; Roswell Park Cancer Institute, United States of America

## Abstract

We have recently isolated novel IFN-inducible gene, Gene associated with Retinoid-Interferon-induced Mortality-1 (*GRIM-1*), using a genetic technique. Moderate ectopic expression of GRIM-1 caused growth inhibition and sensitized cells to retinoic acid (RA)/IFN-induced cell death while high expression caused apoptosis. GRIM-1 depletion, using RNA*i*, conferred a growth advantage. Three protein isoforms (1α, 1β and 1γ) with identical C-termini are produced from *GRIM-1* mRNA. We show that GRIM-1 isoforms interact with NAF1 and DKC1, two essential proteins required for box H/ACA sno/sca RNP biogenesis and suppresses box H/ACA RNA levels in mammalian cells by delocalizing NAF1. Suppression of these small RNAs manifests as inefficient rRNA maturation and growth suppression. Interestingly, yeast Shq1p also caused growth suppression in mammalian cells. Consistent with its growth-suppressive property, GRIM-1 expression is lost in a number of human primary prostate tumors. Our observations support a recent study that *GRIM-1* might act as a co-tumor suppressor in the prostate.

## Introduction

The IFN family of cytokines suppress abnormal cell growth by two mechanisms: 1) directly activating growth arrest/apoptosis in the neoplastic cells [1] and 2) shaping the immune response against neoplastic cells [2]. Among the known direct effects of IFNs on mRNA are - 1) degradation by 2′,5′A-dependent RNaseL [3]; 2) inhibition of cap-dependent translation initiation by PKR *via* phosphorylation of eIF-2α; 3) sequestration of eIF3 by p56 [4] and 4) mTOR-dependent phosphorylation of cap-binding factor eIF4E-BP1 [5]. We and others have shown that combination of IFNs with cell-differentiating agents such as retinoic acid (RA) effectively suppresses tumor growth in clinical and experimental models [1]. To understand the molecular mechanisms that regulate these processes, we employed a genome-wide knockdown method and isolated *GRIM*s [6], [7]. One such novel gene product GRIM-1, suppressed cell growth [8]; and exhibited similarity (∼53%) to a yeast protein, Shq1p. Here, we describe a mechanism by GRIM-1 that controls mature rRNA levels and cell growth.

The synthesis and maturation of rRNA and its assembly with ribosomal proteins plays a central role in regulating cell growth. A hallmark observed in all cancers relate to gain of ribosome function resulting in high synthetic capacities [9], [10], while defect(s) still contribute to pathological conditions as seen in Diamond Blackfan anemia [11] and Dyskeratosis Congenita (DC) [12] that often result in bone marrow failure without affecting other tissues. In addition, diverse several cellular signals/stress pathways appear to control the activity of ribosome assembly and function [13]–[15]. Thus, a comprehensive analysis of these pathways will improve our current understanding of gain, loss and/or mutations as has been recently shown for breast [16] and prostate [17] cancers to design effective therapeutic strategy.

Maturation of rRNA requires at least hundred or more trans-acting protein factors, in addition to the ribosomal proteins, and numerous nucleotide modifications. A group of small non-coding RNAs, the H/ACA RNAs, serves as pseudouridylation guides in assisting dyskerin (DKC1; also known as Nap57 in rodents and Cbf5p in yeast) to catalyze such modification. GRIM-1 (also known as SHQ1 in mammals and Shq1p in yeast) and NAF1 (Naf1p in yeast) are factors that determine and/or regulate the stability of DKC1. In budding yeast, Shq1p depletion resulted in poor growth due to a problem in rRNA maturation [18]. It forms complexes with other proteins *viz.*, Naf1p [19] and a pseudouridine synthetase (Cbf5p) [20] that are required for the biogenesis of small nucleolar (sno) ribonucleoprotein particles (RNPs) (snoRNPs), small Cajal body (sca) RNPs and telomerase RNA component (terc) RNPs required for rRNA maturation, spliceosomal RNA (snRNA) modifications and telomerase activity, respectively. A role for NAF1 and/or DKC1 in box H/ACA RNP biogenesis in mammalian cells have been described [21]–[23]. We show here that human GRIM-1 suppresses cell growth by sequestering NAF1, leading to a loss of box H/ACA RNAs and mature rRNA levels. We also show that yeast Shq1p acts as a growth suppressor in GRIM-1-depleted human cells. These studies uncovered a novel regulatory pathway that controls cell growth by limiting rRNA maturation. The biological relevance of our observations is further attested by a loss of GRIM-1 expression in primary human prostate tumors. In this manuscript we use a) GRIM-1 in our experiments, b) *SHQ1*/SHQ1 when referring to published articles on the mammalian gene/protein and c) *shq1*/Shq1p when referring to the yeast gene/protein.

## Materials and Methods

### Plasmids and Cell lines

Human *GRIM-1* isoforms (α, β and γ) in pCXN_2_-Myc and pEGFP-C2 vectors which express GRIM-1 as Myc and GFP-tagged proteins at C and N termini, respectively have been described [8]. The yeast *shq1* ORF (Open Biosystems) was sub-cloned into pCXN_2_-FLAG vector, to express as an N-terminally FLAG-tagged protein. pLVX-Puro vector (Clontech) was used to produce viral particles expressing GRIM-1 as per our previous studies [24]. Stable cell lines with the indicated gene, either in MCF-7 and/or HeLa (available from ATCC) were generated after selecting with appropriate drug for 1 week to obtain colonies. Colonies (n = 50–75) were pooled and propagated further to avoid a clonal bias in experiments. Cell growth was measured using a colorimetric assay described earlier [25]. All work involving recombinant DNA and/or viral vectors were approved by the Institutional Biosafety Committee of the University of Maryland Baltimore (No. IBC-00000875).

### RNA*i*

A commercially available lentiviral vector (pLKO1) carrying a shRNA capable of targeting human *GRIM-1* (Open Biosystems) was used for depleting endogenous GRIM-1 levels. An empty vector and/or scrambled-shRNA control were used for demonstrating the specificity of knockdown. Stable cell lines were generated after transducing with lentivirus. A non-targetable (RNA*i*-resistant) variant of *GRIM-1* was generated using PCR (Table S1) and expressed from pCXN_2_-Myc vector.

### Antibodies and other reagents

Antibodies specific for Myc-tag (Cell Signaling), FLAG-tag and actin (Sigma-Aldrich), GRIM-1 (developed in our lab), DKC1 (Santa Cruz Biotechnology), NAF1 (from Dr. Yves Henry, CNRS, France), HRP-conjugated anti-mouse/rabbit IgG (GE Healthcare), Alexa Flour® 488-conjugated anti-mouse IgG (Invitrogen), Cy3-conjugated anti-rabbit IgG (Jackson ImmunoResearch Laboratories Inc) were used in Western blot (WB), Immunoprecipitation (IP) and Immunofluorescence analyses in conjunction with recombinant human IFN-β (Biogen, Inc), RA, protease-inhibitor cocktail, DAPI (Sigma-Aldrich) and ECL reagents (Pierce) as in our earlier studies [8], [24].

### Quantitative reverse transcription PCR (qPCR)

Total RNA was employed for cDNA synthesis with gene-specific primer mix (Table S1) using a commercially available kit (Promega). The resultant cDNA was used as template for real-time PCR with SYBR green dye (Sigma-Aldrich, Inc) and gene-specific primers (Table S1). Relative levels of different small RNAs were normalized to those of snRNA-U6. For rRNA processing, the Ct value obtained using primers 6/1 and F/A represent the total 18S and 28S rRNA (mature and unprocessed), respectively, present in the preparation. The Ct values obtained using two different primer sets (4/5 and 2/3) for 5′ETS-18S and (D/E and B/C) for ITS2-28S represent unprocessed rRNA present in the preparation. Fraction of unprocessed rRNA was calculated using the averages of primer pairs 4/5 (unprocessed) over 6/1 (total) and primer pairs 2/3 (unprocessed) over 6/1 (total) for 18S rRNA while primer pairs D/E (unprocessed) over F/A (total) and primer pairs B/C (unprocessed) over F/A (total) for 28S rRNA. For additional information on primers refer Table S1. At least triplicate reactions per primer pair for each sample were employed in each experiment. Each experiment was repeated with three separate batches of RNA and subjected to Student's *t*-test to determine statistical significance of the differences.

### Ribonucleoprotein (RNP)-Immunoprecipitation (IP) (RNP-IP) assay

Cells (2×10^6^) were incubated in medium with 2% paraformaldehyde for 15 min and washed thrice with PBS before lysis using buffer (20 mM HEPES pH 7.0 containing 150 mM NaCl, 1% Triton X-100, 0.1% SDS and 0.1% sodium deoxycholate, 2 mM MgCl_2_). Lysates were centrifuged to remove debris and supernatants were incubated with rabbit anti-Dyskerin IgG (Santa Cruz)) or with an isotypic IgG control at 4°C overnight. Protein A/G-sepharose was added to pull down anti-Dyskerin or control IgG. The pellet was washed twice with the above buffer; bound RNA was extracted, analyzed by real-time PCR for abundance. RNA directly extracted from the same number of cells was used as a control in this experiment to assess their yield in RNP-IP.

## Results

### GRIM-1 suppresses cell growth

**Figure 1:**
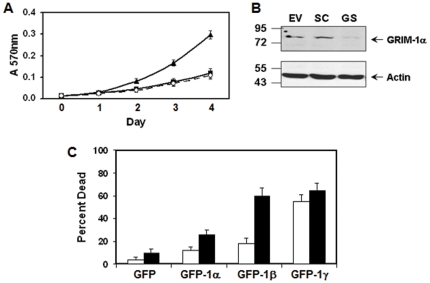
GRIM-1 is a novel growth suppressor. A) GRIM-1 depletion confers a growth advantage during RA/IFN stimulation. Top: Growth of HeLa cells stably expressing pLKO1 (EV; ○) or scrambled shRNA (SC; ▪) or GRIM-1-specific (GS; ▴) shRNA were monitored for 4 days in the presence of a sub-lethal but growth-inhibitory concentration of hIFN-β (1000 U/ml) and RA (1 µM). GRIM-1-depleted cells continue to grow while the others are inhibited by RA/IFN. B) Depletion of GRIM-1 ascertained by WB analysis using GRIM-1-specific antibody and actin-specific antibody was used as loading control. GRIM-1 in GS cells represent ∼12% compared to levels in EV and/or SC when normalized to corresponding actin levels. C) GRIM-1 over-expression sensitizes cells to RA/IFN-induced cell death. HeLa cells transiently expressing GFP-tagged GRIM-1 isoforms (Open bars) were challenged with hIFN-β (2000 U/ml) and RA (2.5 µM) (Solid bars) for 12 h. Change in cell/nuclear appearance of GFP-positive cells was used as a measure of cells undergoing apoptosis. Numbers to the side in WB indicate positions of protein markers.

To ascertain our earlier observation that anti-sense *GRIM-1* conferred a growth advantage in the presence of RA/IFN [8], we employed RNA*i*. For this experiment, we depleted GRIM-1 using lentiviral vectors coding for a *GRIM-1*-specific shRNA, and monitored growth in the presence of RA/IFN. Cells expressing an empty vector and/or scrambled shRNA were used as controls. RA/IFN robustly suppressed the growth of cells expressing either empty vector or scrambled shRNA (Figure 1A). In contrast, cells expressing *GRIM-1*-specific shRNA continued to grow in presence of RA/IFN. Depletion of GRIM-1 was confirmed by performing a WB analysis with a mouse monoclonal antibody that detects full-length GRIM-1 (Figure 1B). GRIM-1 levels were ∼12% in presence of specific shRNA compared to the empty vector and/or scrambled shRNA controls.

The *GRIM-1* mRNA codes for 3 isoforms *viz.*, GRIM-1α, GRIM-1β and GRIM-1γ with identical C-termini [8]. In a complementary experiment, HeLa cells transfected with GFP-tagged GRIM-1 isoforms were challenged with RA/IFN and apoptosis was (shrinkage/chromatin condensation/fragmentation), monitored as earlier [8]. Apoptosis was highest in GRIM-1γ-expressing cells followed by GRIM-1β and GRIM-1α. However, in the presence of RA/IFN, 3.5-fold increase in dying cells was evident in GRIM1β-expressing cells followed by 2-fold increase in GRIM-1α-expressing cells and only modest increase in GRIM-1γ-expressing cells (Figure 1C). Thus, GRIM-1 appears to be a novel growth suppressor and/or apoptosis inducer.

### GRIM-1 interacts with NAF1

**Figure 2:**
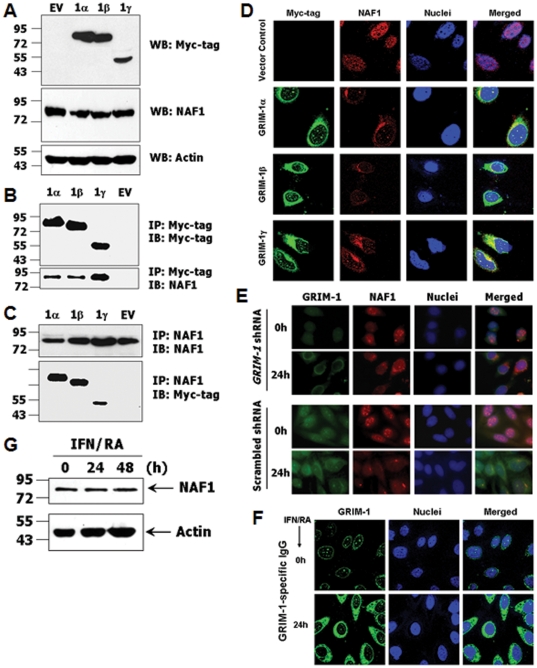
GRIM-1 isoforms interact with endogenous NAF1 in mammalian cells. HeLa cells transiently expressing Myc-tagged GRIM-1 isoforms were analyzed for interaction with NAF1 using the indicated antibodies. A) Expression levels of GRIM-1 isoforms (top), NAF1 (middle) and actin (bottom) in the indicated transfectants. GRIM-1γ levels are low compared to GRIM-1α/β when normalized to actin levels. No significant change in NAF1 levels are observed upon GRIM-1 expression. B & C) Total cell lysates (signal-normalized lysate amount) were subjected to IP with the indicated antibodies and subsequently analyzed by WB with the indicated antibodies. All GRIM-1 isoforms interact with NAF1. More NAF1 in GRIM-1γ-expressing cells is due to more lysate used for IP. D–E) GRIM-1 and NAF1 co-localize *in situ*. D) Confocal images of Myc-tagged GRIM-1 isoforms (green channel) and NAF1 (red channel). Nuclei (blue channel) were visualized using DAPI. GRIM-1 isoforms and NAF1 complexes (yellow) can be seen in the merged channel indicative of interaction *in situ*. Lobed nuclei appear with a high frequency in GRIM-1γ-expressing cells. E) GRIM-1 is necessary for delocalizing NAF1. Immunofluorescent images of GRIM-1 and NAF1 in the indicated cell lines at steady state and RA/IFN-stimulated state. In both cell lines, NAF1 is nuclear at steady state. Upon RA/IFN stimulation, NAF1 is delocalized in SC cells, but not in GS cells. F) Confocal images of endogenous GRIM-1. RA/IFN stimulation increases GRIM-1 that accumulates more in the cytoplasm. G) NAF1 levels are not influenced by IFN/RA treatment. Total cell lysates were prepared at the indicated time point after RA/IFN treatment and analyzed using the indicated antibodies in WB. Numbers to the side in WB indicate positions of protein markers.

Since yeast Shq1p has been suggested to form a complex with Naf1p and assist in the biogenesis of box H/ACA RNA/RNP [18], we wanted to know if such interaction occurs in mammalian cells. To address this, we transiently expressed Myc-tagged GRIM-1 isoforms in HeLa cells and determined its interaction with endogenous NAF1. First, expression of the individual GRIM-1 isoforms was ascertained by performing a WB analysis with Myc-tag-specific antibody (Figure 2A). NAF1 levels were comparable (Figure 2A) under these conditions. However, expression of GRIM-1γ was lower compared to GRIM-1α/β when normalized to actin signals (Figure 2A). Hence, we performed IP using signal-normalized cell lysates using Myc-tag-specific antibody followed by WB analysis with NAF1-specific antibody. All three GRIM-1 isoforms pulled down NAF1 efficiently as revealed by WB analysis of the IP products (Figure 2B). The higher NAF1 signals observed in GRIM-1γ-expressing cells compared to GRIM-1α/β-expressing cells, is not due to higher expression of NAF1 in these lysates, rather it is due to a higher lysate input. This blot was stripped and reprobed with a Myc-tag-specific antibody to exclude differences in the levels of immunoprecipitated GRIM-1 isoforms (Figure 2B). These interactions were further confirmed by a reciprocal IP, where a NAF1-specific antibody was used to pull down Myc-tagged GRIM-1 isoforms (Figure 2C). These experiments for the first time show that GRIM-1 isoforms associate with NAF1.

### GRIM-1 delocalizes NAF1 from Nucleus

We next determined if GRIM-1 isoforms and NAF1 proteins co-localize *in situ*. To address this, we expressed Myc-tagged GRIM-1 isoforms in HeLa cells and tracked NAF1 using confocal microscopy. In control cells, NAF1 was exclusively nuclear with certain focal regions of intense staining (Figure 2D). In contrast, most NAF1 was found outside the nucleus and a majority of it superimposed with GRIM-1 isoforms (Figure 2D). To determine the effects on endogenous proteins, we treated GRIM-1-depleted HeLa cells with RA/IFN and tracked NAF1. Very little NAF1 was present in nuclei of RA/IFN-treated control cells (SC), while GRIM-1-depleted cells (GS) had higher levels of nuclear NAF1 (Figure 2E). This is consistent with an increase in GRIM-1 levels upon RA/IFN treatment that accumulates more in the cytoplasm (Figure 2F). Thus, GRIM-1 delocalizes NAF1. No such effect on the nuclear localization of p300 was noted (data not shown).

To determine if RA/IFN, which induced GRIM-1, affected NAF1 levels, RA/IFN-treated HeLa cell lysates were subjected to WB with NAF1-specific antibody. No discernible change in NAF1 level occurred (Figure 2G). A comparable protein loading was ascertained by re-probing these blots with an actin-specific antibody (Figure 2G). On a similar line, overexpression of GRIM-1 isoforms did not alter NAF1 levels (see Figure 2A).

### GRIM-1 downregulates H/ACA RNAs

**Figure 3:**
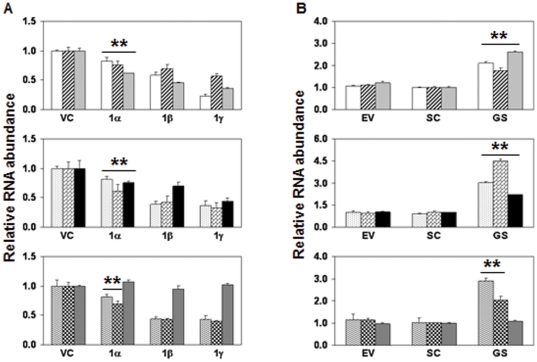
GRIM-1 regulates small non-coding RNA levels. Relative abundance was calculated with respect to spliceosomal RNA (snRNA-U6) levels by real-time PCR. A) Levels of the indicated RNA in HeLa cells stably expressing Myc-tagged GRIM-1 isoforms. GRIM-1 isoforms differentially suppress box H/ACA-containing RNA. Top: snoRNA-73 (open), -74 (hatched), -50 (light gray); Middle: scaRNA-8 (stippled), -13 (brick), TERC (black); Bottom: spliceosomal RNA (snRNA-U2 (wavy)) and box C/D-containing RNA (snoRNA-U3 (checkered)) levels. A related box C/D snoRNA-U13 (dark gray) levels are not influenced by GRIM-1. B) Levels of the indicated RNA upon GRIM-1 depletion in HeLa cells. GRIM-1-depleted cells (GS) show increased box H/ACA-containing RNA. Top: snoRNA-73, -74 and -50; Middle: scaRNA-8, -13, TERC; Bottom: snRNA-U2 and snoRNA-U3 levels. A related box C/D snoRNA-U13 levels is not influenced by GRIM-1. Bar patterns same as in (A). **, *p*<0.01.

Since NAF1 was required for box H/ACA RNP biogenesis in mammalian cells [21], [22], the impact of GRIM-1-mediated NAF1 delocalization would be detrimental to cells. To address this, the levels of all three classes of small non-coding RNA that have box H/ACA motif in addition box C/D snoRNA and spliceosomal RNAs were analyzed by qPCR in cells expressing GRIM-1 isoforms. Indeed, the levels of box H/ACA (sno, sca and terc) RNAs were significantly suppressed (*p*<0.05) in presence of GRIM-1 compared to controls (Figure 3A). Interestingly, some of the spliceosomal RNA (snRNA-U2) and box C/D snoRNA-U3 levels were also significantly (*p*<0.05) suppressed in these cells (Figure 3A). GRIM-1γ, GRIM-1β and GRIM-1α had high, intermediary and lowest inhibitory effect, respectively, on these small RNAs. The levels of box C/D snoRNA-U13 were comparable in cells expressing different GRIM-1 isoforms (Figure 3A). We next examined the effect of GRIM-1 depletion on these small RNAs. We employed two cell lines expressing either an empty vector or a scrambled shRNA as controls. Significantly (*p*<0.01), higher levels of these small RNAs were present in GRIM-1-depleted cells compared to controls (Figure 3B). The levels of box C/D snoRNA-U13 was unaffected (Figure 3B) under these conditions.

### Restoration of GRIM-1 expression in GRIM-1-depleted cells downregulates H/ACA RNAs

**Figure 4:**
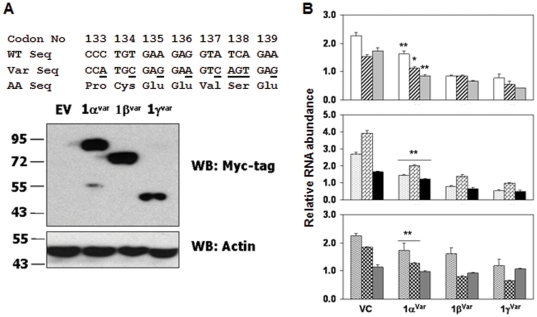
GRIM-1 regulates small non-coding RNA levels. A) Construction of shRNA-resistant *GRIM-1* variant. Top: *GRIM-1* shRNA-targeted region showing the wild-type sequence (WT Seq) and their corresponding amino acids (AA Seq) number (Codon No). Base changes coding for the same amino acids are underlined in the variant sequence (Var Seq). Bottom: *GRIM-1* shRNA does not target the variant sequence for silencing. Expression of Myc-tagged GRIM-1^Var^ isoforms in GRIM-1-depleted cells. Actin was used as a loading control. B) The repressive effect of GRIM-1 on small non-coding RNA reappears upon GRIM-1^Var^ expression in GRIM-1-depleted cells (GS). Levels of the indicated RNA in HeLa cells stably expressing Myc-tagged GRIM-1^Var^ isoforms. GRIM-1^Var^ isoforms differentially suppress snoRNA-73, -74, -50, scaRNA-8, -13, TERC, snRNA-U2 and snoRNA-U3 levels. A related box C/D snoRNA-U13 levels are not influenced by GRIM-1. Relative abundance was calculated with respect to spliceosomal RNA (snRNA-U6) levels by real-time PCR. *, *p*<0.01; **, *p*<0.001. Numbers to the side in WB indicate positions of protein markers. Bar patterns same as in Figure 3.

Since RNA*i* may some time cause off-target effects [26], [27] and RNA*i*-mediated GRIM-1 depletion does not distinguish the effects of individual GRIM-1 isoforms, we next performed reconstitution experiments. Since, the *GRIM-1*-specific shRNA targets a region within the ORF, it will also target the transgene-derived mRNA for degradation/inhibition. Hence, we designed a variant that codes for wild-type amino acids in the shRNA-targeted region (Figure 4A) that was resistant to degradation/inhibition by the resident *GRIM-1* shRNA. After ensuring the expression of variant GRIM-1, in GRIM-1-depleted cells (Figure 4A), we quantified the levels of small RNAs. Small RNA levels were significantly (*p*<0.05) suppressed by the individual variant GRIM-1 isoforms compared to the parent cell line (Figure 4B); in a manner similar to overexpressed GRIM-1 isoforms (see Figure 3). The levels of box C/D snoRNA-U13 did not differ in cells expressing variant GRIM-1 isoforms under these conditions (Figure 4B).

### GRIM-1 represses rRNA processing

**Figure 5:**
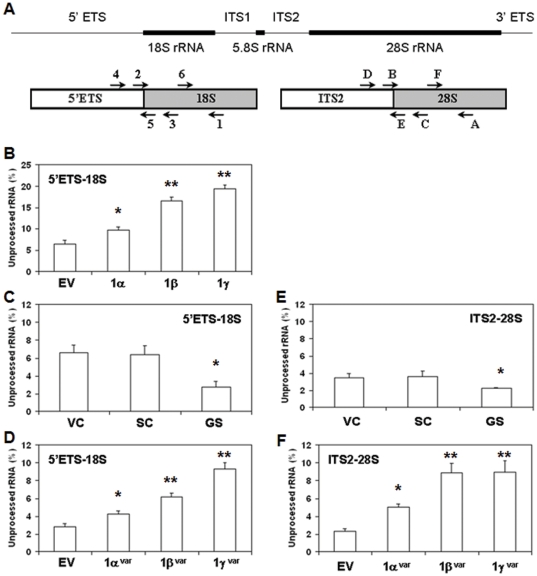
GRIM-1-mediated effects on rRNA processing. A) A Modular representation of mammalian rRNA primary transcript. Top: Relative positions of regions absent (thin lines) and present in the mature rRNA (solid lines). Bottom: Relative positions of primers used to analyze rRNA processing. Primer (1 or A) is used for first-strand synthesis in RT. The Ct value obtained using primers 6/1 and F/A represent the total rRNA (mature and unprocessed) present in the preparation. The Ct value obtained using two different primer sets (4/5 and 2/3) for 5′ETS-18S and (D/E and B/C) for ITS2-28S represent unprocessed rRNA present in the preparation. Primers 2 and 5 cannot amplify when 18S rRNA has matured. Similarly, primers B and E cannot amplify when 28S rRNA has matured. B) GRIM-1 isoforms suppress rRNA processing. Isoform-specific inhibitory effects are evident on 5′ETS-18S region with GRIM-1γ being a potent inhibitor of processing and GRIM-1α is the least inhibitory. The values represent the averages of primer pairs 4/5 (unprocessed) over 6/1 (total) and primer pairs 2/3 (unprocessed) over 6/1 (total) for 18S rRNA. C) Efficient processing of 18S rRNA regions in GRIM-1-depleted cells (GS) compared to empty vector control (pLKO1; EV) and/or scrambled shRNA control (SC). Values were calculated as mentioned above. D) Reappearance of the inhibitory effect on rRNA processing upon GRIM-1^Var^ expression in GRIM-1-depleted cells (GS). Isoform-specific effects are evident on 5′ETS-18S region. E) Efficient processing of 28S rRNA regions in GRIM-1-depleted cells (GS) compared to empty vector control (pLKO1; EV) and/or scrambled shRNA control (SC). The values represent the averages of primer pairs D/E (unprocessed) over F/A (total) and primer pairs B/C (unprocessed) over F/A (total) for 28S rRNA. F) GRIM-1^var^ isoforms show inhibitory effect on ITS2-28S region. GRIM-1β^var^ and GRIM-1γ^var^ exert similar inhibitory effect while GRIM-1α^var^ is less inhibitory on ITS2-28S rRNA processing. *, *p*<0.01; **, *p*<0.001.

SnoRNA-directed modification and cleavage of the primary rRNA transcript yields mature 18S, 5.8S and 28S rRNA and is critical for the formation of mature ribosomes. Therefore, we next quantified the effect of GRIM-1 isoforms on rRNA processing, using primers that can distinguish the precursor and mature human rRNA molecules. One of the primer annealing site overlaps the processed region (junction-specific) and another further downstream in the invariant region representing unprocessed and total rRNA, respectively (Figure 5A). Since, an ordered processing in the 5′ETS region is vital for subsequent cleavages to generate mature 18S rRNA, we analyzed 5′ETS-18S rRNA junction in cell lines expressing GRIM-1 isoforms. The invariant portion of 18S served as the internal control in this analysis. A similar strategy was used for analyzing 28S rRNA processing. Raw data from the junction-specific primer was normalized using invariant primer region and represented as fraction rRNA unprocessed. As shown in Figure 5B, in presence of GRIM-1 isoforms the level of unprocessed rRNA was higher compared to the control cell line. Under these conditions, GRIM-1γ was the most potent inhibitor of 5′ETS-18S rRNA processing followed by GRIM-1β and GRIM-1α. In GRIM-1-depleted cells, 5′ETS-18S junction was more efficiently processed than in the controls (Figure 5C). Upon reconstitution of variant GRIM-1 isoforms into GRIM-1-depleted cells, the inhibitory effect on 5′ETS-18S rRNA processing reappeared (Figure 5D). As noted with 5′ETS-18S region, GRIM-1 depletion increased processed ITS2-28S rRNA compared to the controls (Figure 5E). Upon reconstitution of variant GRIM-1 isoforms, the inhibitory effect on ITS2-28S rRNA reappeared showing isoform-specific differences to a certain extent (Figure 5F). In this assay, GRIM-1β and GRIM-1γ inhibited ITS2-28S processing equivalently (Figure 5F).

### GRIM-1 interacts with Dyskerin in mammalian cells

**Figure 6:**
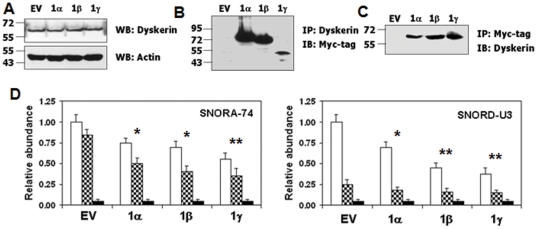
GRIM-1 isoforms interact with endogenous Dyskerin in mammalian cells. HeLa cells transiently expressing Myc-tagged GRIM-1 isoforms were analyzed for interaction with Dyskerin using the indicated antibodies. A) Expression levels of Dyskerin (top) and actin (bottom) in the indicated transfectants. No significant change in Dyskerin levels are observed upon GRIM-1 expression. B & C) Total cell lysates (signal-normalized lysate amount) were subjected to IP with the indicated antibodies and subsequently probed with the indicated antibodies. All GRIM-1 isoforms interact with Dyskerin. D) Quantification of Dyskerin-bound RNA in the indicated cell lines. RNA extracted following RNP-IP of cross-linked cell lysates using Dyskerin-specific antibody, was used in RT and analyzed by real-time PCR using primers. Dyskerin-bound RNA levels are low in GRIM-1-expressing cells. Majority of snoRNA-74 fraction is bound by Dyskerin (a box H/ACA core protein) while only a minor fraction of snoRNA-U3 is bound by Dyskerin. Open bars represent RNA levels (total) in cells, Checkered bars represent Dyskerin-bound RNA levels in cells and Solid bars represent RNP-IP performed using an IgG control. *, *p*<0.01; **, *p*<0.001. Numbers to the side in WB indicate positions of protein markers.

While these studies were in progress, a report demonstrating the binding of SHQ1 to DKC1 *in vitro* [28] appeared. Therefore, we tested whether GRIM-1 can bind DKC1 in mammalian cells. DKC1 levels were very similar in vector- and GRIM-1 isoform-expressing cells (Figure 6A). We next performed IP and WB analyses for assessing the interactions between DKC1 and GRIM-1. Dyskerin and GRIM-1 isoforms interacted with each other in a manner similar to NAF1 (Figures 6B & C).

### Box H/ACA RNA-bound Dyskerin levels are low in GRIM-1-expressing cells

In light of the interactions between NAF1-GRIM-1 and DKC1-GRIM-1, we hypothesized DKC1 to be delocalized as the core trimer requires NAF1 to be functional. To address this, we resorted to RNP-IP, which would address whether DKC1 associated with nascent box H/ACA RNA. DKC1-specific antibody was used in RNP-IP of cross-linked lysates from GRIM-1 isoform-expressing cells. RNA recovered from the IP products was quantified using real-time PCR. These analyses indicated that a pool of DKC1 was devoid of box H/ACA RNAs (Figure 6D) in the presence of GRIM-1 isoforms.

### Yeast Shq1p exerts GRIM-1α-like effects in mammalian cells

**Figure 7:**
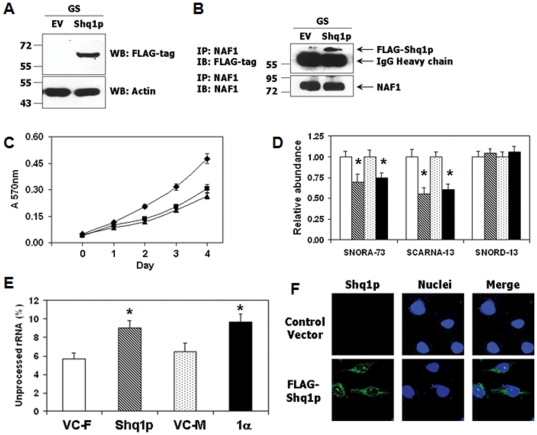
Yeast Shq1p protein exhibits GRIM-1α-like behavior in mammalian cells. A) Expression of FLAG-tagged Shq1p in GRIM-1-depleted HeLa cells ascertained by WB. B) Shq1p interacts with endogenous NAF1. IP and WB analysis were performed as described previously with the indicated antibodies. C) Growth assay of empty vector control (VC-F; ♦), Shq1p-expressing (▪) and GRIM-1α-expressing (▴) cells. D) Box H/ACA RNA levels are suppressed by Shq1p and GRIM-1α to a similar extent. Bar patterns are: pCXN_2_-FLAG vector (Open; VC-F), FLAG-Shq1p (Hatched; Shq1p), pCXN_2_-Myc vector (Stippled; VC-M) and Myc-tagged GRIM-1α (Solid; 1α). E) Inhibition of 18S rRNA processing by Shq1p and GRIM-1α are similar. Bar patterns as in D. The values represent the averages of primer pairs 4/5 (unprocessed) over 6/1 (total) and primer pairs 2/3 (unprocessed) over 6/1 (total) for 18S rRNA. See Fig. 5 legend for additional information. F) Over-expressed Shq1p more accumulates in the cytoplasm. Confocal images showing more Shq1p in the cytoplasm compared to the nucleus. *, *p*<0.01. Numbers to the side in WB indicate positions of protein markers.

Since Shq1p was critical for viability and growth in yeast, we next determined how it behaved in human cells. To address this, we expressed N-terminally FLAG-tagged Shq1p in GRIM-1-depleted HeLa cells (Figure 7A) and performed an IP analysis to detect its interaction with endogenous NAF1. Indeed, Shq1p interacted with NAF1 (Figure 7B). However, to our surprise, Shq1p caused growth inhibition (Figure 7C) like GRIM-1α. This observation prompted us to analyze steady-state levels of box H/ACA RNA in Shq1p-expressing cells. GRIM-1α-reconstituted cells were used as a positive control for this experiment. Indeed, box H/ACA RNA levels were suppressed very similarly by Shq1p and GRIM-1α (Figure 7D) compared to their respective controls. Next, we analyzed 18S rRNA processing in these two cell lines. The levels of unprocessed 18S rRNA increased significantly (*p*<0.05) in Shq1p- and GRIM-1α-expressing cells (Figure 7E). As observed with GRIM-1, Shq1p also accumulated more in the cytoplasm (Figure 7F).

### GRIM-1 expression is lost in human primary tumors

**Figure 8:**
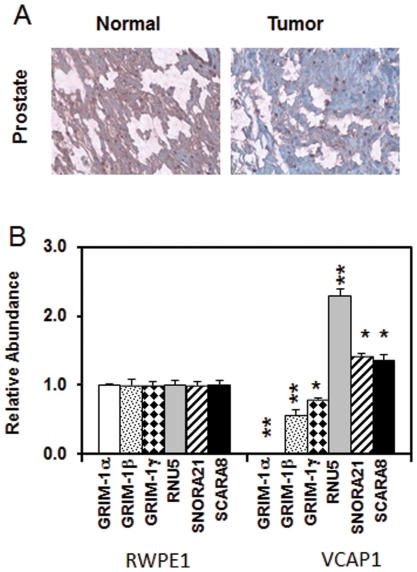
GRIM-1 expression is lost in prostatic epithelial cells. A) NCI-TARP array was analyzed by immunohistochemistry as per standard procedures. A representative patient-matched sample showing low expression of GRIM-1 (brown signals) in prostate tumors compared to normal prostate. See Table 1 for additional information. B) *GRIM-1* mRNA levels are low in VCaP compared to RWPE-1 as analyzed by qPCR while the other indicated RNAs are high in VCaP compared to RWPE-1 cell line. *GRIM-1* mRNA levels were analyzed using three different primer pairs (Table S3). *, *p*<0.01; **, p<0.001.

To determine the pathologic relevance of GRIM-1, we subjected the NCI-TARP human tissue array to immunohistochemical analysis with a mouse GRIM-1-specific antibody (Figure 8A). Based on the intensity of GRIM-1 signals (low to high) we categorized them arbitrarily into five groups. The prostate specimens had normal (n = 38) and tumor (n = 46) tissues, among which 24 were patient-matched. Statistical analysis of the prostate tumors indicated that 100% of the samples showed loss/reduction compared to their normal tissues with severe loss/reduction observed in 75% of the samples (Table 1). These observations suggest a potential tumor suppressor-like function for GRIM-1. These data are also consistent with our recent demonstration of GRIM-1 isoforms suppress tumor xenograft growth in mice [8].

**Table 1:** GRIM-1 expression levels in normal and tumor specimens analyzed by immunohistochemistry.

Level@ →	Normal						Tumor						Loss (%)
	+	++	+++	++++	+++++	Total	+	++	+++	++++	+++++	Total	
Prostate	4	5	7	14	8	**38**	21	17	8			**46**	
Prostate (paired)	3	3	4	8	6	**24**	7	11	6			**24**	**75**

@Expression level graded on an arbitrary scale (%) using signal ratio of GRIM-1 to DAPI.

+ (0–20) – Severe loss, ++ (21–40) – Significant loss, +++ (41–60) – Moderate loss, ++++ (61–80) – Slight loss, +++++ (81–100) – No loss.

We further examined *GRIM-1* in patient-matched prostate specimens of 4 patients (NCI CHTN) that had tumor content from 40% to 80%. All patients in this sample had Stage III–IV disease. Since *GRIM-1* spans ∼100-kb region, we employed primers to amplify the 11 individual exons (Table S2). Except for exon 2, we were able to amplify all exons and found no point mutations (data not shown). Because we could not get RNAs from these tumors, we next determined *GRIM-1* transcript levels in a non-oncogenic prostate epithelial cell line RWPE-1 and a metastatic prostate tumor cell line VCaP using qPCR. As shown in Figure 8B, there was a significant reduction in the levels of *GRIM-1* isoform-specific ORF regions (Table S3) in VCaP compared to RWPE-1. The GRIM-1α transcript level was extremely low in the tumor cell line. Consistent with these observations, expression levels of *RNU5*, *SNORA21* and *SCARNA8* were significantly (p<0.05) high in VCaP compared to RWPE1.

## Discussion

GRIM-1 is a novel IFN-induced growth regulator [8]. IFNs are known to regulate a number of post-transcriptional processes that modulate several cellular responses [29]. Two well-studied IFN-inducible proteins *viz.*, PKR and RNaseL affect protein synthesis and mRNA stability, respectively. Activated PKR phosphorylates eIF-2α leading to an inhibition of cap-dependent translational initiation [30]–[32]. The IFN-inducible OAS, which produce 2′, 5′-linked oligoadenylates [33], [34] activate a latent endoribonuclease, RNaseL, which cleaves certain cellular RNAs to regulate cellular and viral growth [3], [35]. A third IFN-inducible protein, p56 inhibits translation by associating with eIF-3 [4]. IFN-induced mTOR signaling pathway suppresses protein translation *via* phosphorylation of eIF4E-BP. In this study, we defined another novel IFN-inducible mechanism that regulates cell growth by controlling RNA metabolism. Maturation of rRNA is controlled by snoRNPs, a group of small non-coding RNA-containing nucleolar protein complexes. We described here how *GRIM-1* [8] controls this process at the stage of snoRNP biogenesis, by delocalization of NAF1, a factor necessary for snoRNP maturation. The mechanism of GRIM-1 action appears to be distinct from that of another IFN-inducible gene product ISG20 [36] although the latter also affected mature rRNA levels.

Our current understanding of rRNA biogenesis is primarily based on literature from the budding yeast. However, the mechanisms that control it in mammalian cells are unclear. In yeast, Shq1p complexes with another protein, Naf1p and both these proteins were required for viability [18]. However, significant differences exist between GRIM-1 and Shq1p: 1) unlike the yeast *shq1*, *GRIM-1* mRNA codes for three isoforms that differentially affect cell growth and rRNA biogenesis; 2) sequence differences between GRIM-1 and Shq1p; and 3) depletion of Shq1p suppressed cell viability. In contrast, depletion of GRIM-1 provided growth advantage in presence of IFN while overexpression caused apoptosis (Figure 1). Despite these functional differences, we found NAF1-GRIM-1 and NAF1-Shq1p physical interactions occur in human cells (Figures 2 & 7). However, both GRIM-1 and Shq1p caused growth suppression upon reconstitution into GRIM-1-depleted human cells. Consistent with a report that Shq1p interacted with another yeast protein Cbf5p [20], we found DKC1 binds to GRIM-1 (Figure 6). Interestingly, SHQ1 interacted only with DKC1 and not with NAF1; while DKC1 bound either SHQ1 or NAF1 and excess NAF1 could displace SHQ1 from DKC1 *in vitro* [28]. Such interpretations were primarily based on *in vitro* translated protein(s).

The N-terminal region of Shq1p appears to have a HSP20-like domain found in co-chaperones of Hsp90 [37]. Interestingly, human and yeast proteins show very high identities in this region. However, a recent *in vitro* reconstitution studies demonstrated that this HSP20-like domain in SHQ1 is dispensable for interactions with DKC1 [28]. For example, GRIM-1β and GRIM-1γ, which lacked this domain, interacted with DKC1 (Figure 6). Since, the HSP20-like domain is not required for stabilizing DKC1 as originally proposed [28], we suggest that GRIM-1 may assist in the opening up the inactive NAF1 homo-dimer, as seen in yeast Naf1p [38], for interaction(s) with DKC1 and NOP10. This is consistent with our observation that GRIM-1γ, which lacks the HSP20-like domain (but found in GRIM-1α) is able to co-IP more NAF1; interact with DKC1 and exert toxic effects. Such low DKC1 levels upregulate tumor suppressor p53 and CDK-inhibitor p21 levels to exert growth suppression and/or apoptosis [39].

Depletion of Shq1p and/or Naf1p caused a severe loss in box H/ACA RNA/RNP levels with concomitant defects in rRNA processing that resulted in poor viability of yeast cells [18]. We found that human GRIM-1 suppressed growth, by repressing box H/ACA RNA levels and rRNA processing. Surprisingly, yeast Shq1p behaved like GRIM-1α (Figure 7) upon its reconstitution in GRIM-1-depleted cells. Thus, sequence differences between these proteins do not appear to account for their functional differences. On a similar note, depletion of SHQ1 in HeLa cells impacted levels of E1 (U17) and E3 H/ACA RNAs far less than DKC1 (NAP57) depletion [28]. Based on these observations we suggest that GRIM-1/Shq1p need not necessarily be a cell survival factor. Many cellular factors including p53 and the MAP kinases of JNK subgroup act in a yin-yang manner in cell growth regulation [40]–[42]. We and others have shown earlier that thioredoxin and thioredoxin reductase which are critical players in cellular redox control, also act as growth suppressors. Thus, depending on the cellular environment GRIM-1 may act as a survival or growth-suppressive factor. Such activities may depend on the post-translational modifications and association(s) with other cellular factor(s), whose identities is/are unclear at this stage. The C-terminal region of GRIM-1 is rich in serine/threonine residues, a feature absent in Shq1p, may explain in part its anti-cellular effects. One clear difference between human GRIM-1 and Shq1p is the absence of caspase-cleavage sites in the latter. Furthermore, yeast does not have a known caspase. Unlike Shq1p, GRIM-1 produces three isoforms. These differences could account for differential behavior of GRIM-1 and Shq1p in their respective species. It is also likely that GRIM-1/Shq1p might interact with some undefined cellular factors (absent in yeast) in mammalian cells to exert growth suppression. Even though, box H/ACA and C/D snoRNA assembles into RNPs with different protein factors, unexpectedly box C/D snoRNA-U3 level was also suppressed by GRIM-1 isoforms (Figures 3 & 4). This observation is similar to earlier reports that documented a drop in C/D snoRNA-U3 (∼45%) levels upon DKC1 depletion [22] and drop in C/D snoRNA-U24 (∼40%) and C/D snR78 (∼25%) levels upon Naf1p depletion [43]. Since, majority of NAF1 molecules were delocalized in presence of GRIM-1 (Figure 2), certain box C/D snoRNA levels *e.g.* U3 may also drop. When this manuscript was under preparation, a report that DKC1 was necessary for cell division but not cell survival appeared. In this study, fibrillarin, the enzymatic component of box C/D RNP, did not localize to nucleoli in DKC1-null cells and such cells could survive for 3 months [39]. This observation further supports our notion that DKC1 may be required for proper localization of, at least some if not all, box C/D RNPs as snoRNA-U3 is detectable in RNP-IPs using DKC1 antibody (Figure 6). On a similar note, DC mutations found in NHP2 and NOP10 affected pre-RNP assembly but not tetramer formation [44], while mutations in DKC1 either weakened or enhanced its association with SHQ1 [44], [45]. Thus, inefficient DKC1 activity appears to be the primary reason for shortened telomeres in individuals with DC; and such effects may be exerted on tercRNP levels by GRIM-1 expression.

In eukaryotes, rRNA and snRNA undergo maturation-associated pseudouridylation mediated by sno and scaRNPs, respectively [46], [47] while box C/D snoRNPs methylate the ribose moieties of specific nucleobases [48], [49]. The number of such modifications increases with the complexity of the organism though the same protein machinery performs the task by increasing its RNA repertoire to target different sites. The precise role of these two modifications is not clear although box C/D and H/ACA RNA expression levels differ in a tissue-specific manner [50]. All eukaryotic box H/ACA sno/scaRNAs require a core protein machinery comprising of four highly-conserved proteins, and mutations in them are rare *e.g. DKC1* [51], *NOP10* [52] and *NHP2* [53]. X-linked DC is caused by mutations in *DKC1*, are characterized by bone marrow failure and increased susceptibility to cancer [12], [54]. DKC1 activity was required for rRNA modifications that mediate IRES-mediated translation initiation of cellular and viral RNAs. Translation of mRNAs coding for XIAP and Bcl-X_L_, were severely affected in *Dkc1^m^* cells [55]. Consistent with its growth-suppressive property, we found that *GRIM-1* expression and/or levels is/are suppressed in human prostate cancers (Figure 8). This observation agrees to our earlier report that *GRIM-1* mRNA levels in cancer cell lines are very low and IFN induced them to varying degrees while over expression of GRIM-1 isoforms suppressed growth of tumor xenografts [8], establishing an important link between rRNA metabolism and tumor growth suppression. Interestingly, chicks imposed with retinal defocus, upregulate SHQ1 levels to stop ocular growth [56]. More importantly, a very recent study reported deletion of *SHQ1* locus in primary prostate cancers [17] suggesting it to be a cooperative tumor suppressor. This observation independently supports our finding that prostate cancers show severe loss of GRIM-1 expression (Figure 8) that attests to a growth-suppressive function in mammals.

Although ribosomal RNA and ribosomes are seen as constitutively active components of pro-growth and pro-survival pathways, a number of recent studies have shown that certain ribosomal proteins act as tumor suppressors [57]. Certain snoRNAs, such as ACA45, have been shown to possess miRNA-like functions and regulate functions beyond their presently perceived notion that they primarily regulate rRNA metabolism [58]. In some cases, low levels of certain snoRNAs such as RNU44, RNU48 and RNU43 have been correlated with poor prognosis of breast and head and neck squamous cell carcinomas [59]. Abnormal ribosome biogenesis has been shown to result in chromosomal instability [60]. An earlier study reported that IFN-β down regulated the expression of RPL23A in some melanoma cells to achieve growth suppression [61]. Thus, IFNs appear to target different components of translational machinery to achieve tumor growth suppression.

Finally, an important question arises as to why eukaryotes need two additional factors for H/ACA RNP biogenesis when archea have none? It was proposed that SHQ1 and NAF1, the assembly factors, act sequentially on DKC1 [28]. From previous studies it is clear that depletion of NAF1/Naf1p has more profound effects on DKC1/Cbf5p levels than does SHQ1/Shq1p depletion [18], [22]. Hence, we believe SHQ1 and NAF1 regulate box H/ACA RNA/RNP levels in a negative and positive manner, respectively in mammals.

## Supporting Information

Table S1 Primers used in this study for RT-PCR, cloning and rRNA processing.
(DOCX)

Table S2 GRIM-1 primers for amplifying exons and sequencing.
(DOCX)

Table S3 GRIM-1 primers for analyzing mRNA regions present in the preparation.
(DOCX)

## References

[1] Kalvakolanu DV (2004). The GRIMs: a new interface between cell death regulation and interferon/retinoid induced growth suppression.. Cytokine Growth Factor Rev.

[2] Dunn GP, Koebel CM, Schreiber RD (2006). Interferons, immunity and cancer immunoediting.. Nat Rev Immunol.

[3] Zhou A, Hassel BA, Silverman RH (1993). Expression cloning of 2-5A-dependent RNAase: a uniquely regulated mediator of interferon action.. Cell.

[4] Guo J, Hui DJ, Merrick WC, Sen GC (2000). A new pathway of translational regulation mediated by eukaryotic initiation factor 3.. Embo J.

[5] Kroczynska B, Kaur S, Platanias LC (2009). Growth suppressive cytokines and the AKT/mTOR pathway.. Cytokine.

[6] Angell JE, Lindner DJ, Shapiro PS, Hofmann ER, Kalvakolanu DV (2000). Identification of GRIM-19, a novel cell death-regulatory gene induced by the interferon-beta and retinoic acid combination, using a genetic approach.. J Biol Chem.

[7] Hofmann ER, Boyanapalli M, Lindner DJ, Weihua X, Hassel BA (1998). Thioredoxin reductase mediates cell death effects of the combination of beta interferon and retinoic acid.. Mol Cell Biol.

[8] Hofmann ER, Nallar SC, Lin L, D'Cunha J, Lindner DJ (2010). Identification and characterization of GRIM-1, a cell-death-associated gene product.. J Cell Sci.

[9] Belin S, Beghin A, Solano-Gonzalez E, Bezin L, Brunet-Manquat S (2009). Dysregulation of ribosome biogenesis and translational capacity is associated with tumor progression of human breast cancer cells.. PLoS One.

[10] Silvera D, Formenti SC, Schneider RJ (2010). Translational control in cancer.. Nat Rev Cancer.

[11] Ohene-Abuakwa Y, Orfali KA, Marius C, Ball SE (2005). Two-phase culture in Diamond Blackfan anemia: localization of erythroid defect.. Blood.

[12] Ruggero D, Grisendi S, Piazza F, Rego E, Mari F (2003). Dyskeratosis congenita and cancer in mice deficient in ribosomal RNA modification.. Science.

[13] Arabi A, Wu S, Ridderstrale K, Bierhoff H, Shiue C (2005). c-Myc associates with ribosomal DNA and activates RNA polymerase I transcription.. Nat Cell Biol.

[14] Grandori C, Gomez-Roman N, Felton-Edkins ZA, Ngouenet C, Galloway DA (2005). c-Myc binds to human ribosomal DNA and stimulates transcription of rRNA genes by RNA polymerase I.. Nat Cell Biol.

[15] Trere D, Ceccarelli C, Montanaro L, Tosti E, Derenzini M (2004). Nucleolar size and activity are related to pRb and p53 status in human breast cancer.. J Histochem Cytochem.

[16] Ding L, Ellis MJ, Li S, Larson DE, Chen K (2010). Genome remodelling in a basal-like breast cancer metastasis and xenograft.. Nature.

[17] Taylor BS, Schultz N, Hieronymus H, Gopalan A, Xiao Y (2010). Integrative genomic profiling of human prostate cancer.. Cancer Cell.

[18] Yang PK, Rotondo G, Porras T, Legrain P, Chanfreau G (2002). The Shq1p.Naf1p complex is required for box H/ACA small nucleolar ribonucleoprotein particle biogenesis.. J Biol Chem.

[19] Ito T, Chiba T, Ozawa R, Yoshida M, Hattori M (2001). A comprehensive two-hybrid analysis to explore the yeast protein interactome.. Proc Natl Acad Sci U S A.

[20] Ho Y, Gruhler A, Heilbut A, Bader GD, Moore L (2002). Systematic identification of protein complexes in Saccharomyces cerevisiae by mass spectrometry.. Nature.

[21] Darzacq X, Kittur N, Roy S, Shav-Tal Y, Singer RH (2006). Stepwise RNP assembly at the site of H/ACA RNA transcription in human cells.. J Cell Biol.

[22] Hoareau-Aveilla C, Bonoli M, Caizergues-Ferrer M, Henry Y (2006). hNaf1 is required for accumulation of human box H/ACA snoRNPs, scaRNPs, and telomerase.. Rna.

[23] Kittur N, Darzacq X, Roy S, Singer RH, Meier UT (2006). Dynamic association and localization of human H/ACA RNP proteins.. Rna.

[24] Sun P, Nallar SC, Kalakonda S, Lindner DJ, Martin SS (2009). GRIM-19 inhibits v-Src-induced cell motility by interfering with cytoskeletal restructuring.. Oncogene.

[25] Skehan P, Storeng R, Scudiero D, Monks A, McMahon J (1990). New colorimetric cytotoxicity assay for anticancer-drug screening.. J Natl Cancer Inst.

[26] Jackson AL, Bartz SR, Schelter J, Kobayashi SV, Burchard J (2003). Expression profiling reveals off-target gene regulation by RNAi.. Nat Biotechnol.

[27] Scacheri PC, Rozenblatt-Rosen O, Caplen NJ, Wolfsberg TG, Umayam L (2004). Short interfering RNAs can induce unexpected and divergent changes in the levels of untargeted proteins in mammalian cells.. Proc Natl Acad Sci U S A.

[28] Grozdanov PN, Roy S, Kittur N, Meier UT (2009). SHQ1 is required prior to NAF1 for assembly of H/ACA small nucleolar and telomerase RNPs.. Rna.

[29] Stark GR, Kerr IM, Williams BR, Silverman RH, Schreiber RD (1998). How cells respond to interferons.. Annu Rev Biochem.

[30] Clemens MJ, Elia A (1997). The double-stranded RNA-dependent protein kinase PKR: structure and function.. J Interferon Cytokine Res.

[31] Thomis DC, Doohan JP, Samuel CE (1992). Mechanism of interferon action: cDNA structure, expression, and regulation of the interferon-induced, RNA-dependent P1/eIF-2 alpha protein kinase from human cells.. Virology.

[32] Gale M, Tan SL, Katze MG (2000). Translational control of viral gene expression in eukaryotes.. Microbiol Mol Biol Rev.

[33] Player MR, Torrence PF (1998). The 2-5A system: modulation of viral and cellular processes through acceleration of RNA degradation.. Pharmacol Ther.

[34] Rebouillat D, Hovanessian AG (1999). The human 2′, 5′-oligoadenylate synthetase family: interferon-induced proteins with unique enzymatic properties.. J Interferon Cytokine Res.

[35] Li XL, Blackford JA, Judge CS, Liu M, Xiao W (2000). RNase-L-dependent destabilization of interferon-induced mRNAs. A role for the 2-5A system in attenuation of the interferon response.. J Biol Chem.

[36] Gongora C, David G, Pintard L, Tissot C, Hua TD (1997). Molecular cloning of a new interferon-induced PML nuclear body-associated protein.. J Biol Chem.

[37] Singh M, Gonzales FA, Cascio D, Heckmann N, Chanfreau G (2009). Structure and functional studies of the CS domain of the essential H/ACA ribonucleoparticle assembly protein SHQ1.. J Biol Chem.

[38] Leulliot N, Godin KS, Hoareau-Aveilla C, Quevillon-Cheruel S, Varani G (2007). The box H/ACA RNP assembly factor Naf1p contains a domain homologous to Gar1p mediating its interaction with Cbf5p.. J Mol Biol.

[39] Ge J, Rudnick DA, He J, Crimmins DL, Ladenson JH (2010). Dyskerin ablation in mouse liver inhibits rRNA processing and cell division.. Mol Cell Biol.

[40] Campisi J (2002). Cancer and aging: yin, yang, and p53.. Sci Aging Knowledge Environ.

[41] Deppert W (1994). The yin and yang of p53 in cellular proliferation.. Semin Cancer Biol.

[42] Weston CR, Davis RJ (2007). The JNK signal transduction pathway.. Curr Opin Cell Biol.

[43] Dez C, Noaillac-Depeyre J, Caizergues-Ferrer M, Henry Y (2002). Naf1p, an essential nucleoplasmic factor specifically required for accumulation of box H/ACA small nucleolar RNPs.. Mol Cell Biol.

[44] Trahan C, Martel C, Dragon F (2010). Effects of dyskeratosis congenita mutations in dyskerin, NHP2 and NOP10 on assembly of H/ACA pre-RNPs.. Hum Mol Genet.

[45] Grozdanov PN, Fernandez-Fuentes N, Fiser A, Meier UT (2009). Pathogenic NAP57 mutations decrease ribonucleoprotein assembly in dyskeratosis congenita.. Hum Mol Genet.

[46] Ganot P, Bortolin ML, Kiss T (1997). Site-specific pseudouridine formation in preribosomal RNA is guided by small nucleolar RNAs.. Cell.

[47] Ni J, Tien AL, Fournier MJ (1997). Small nucleolar RNAs direct site-specific synthesis of pseudouridine in ribosomal RNA.. Cell.

[48] Cavaille J, Nicoloso M, Bachellerie JP (1996). Targeted ribose methylation of RNA in vivo directed by tailored antisense RNA guides.. Nature.

[49] Kiss-Laszlo Z, Henry Y, Bachellerie JP, Caizergues-Ferrer M, Kiss T (1996). Site-specific ribose methylation of preribosomal RNA: a novel function for small nucleolar RNAs.. Cell.

[50] Castle JC, Armour CD, Lower M, Haynor D, Biery M (2010). Digital genome-wide ncRNA expression, including SnoRNAs, across 11 human tissues using polyA-neutral amplification.. PLoS One.

[51] Heiss NS, Knight SW, Vulliamy TJ, Klauck SM, Wiemann S (1998). X-linked dyskeratosis congenita is caused by mutations in a highly conserved gene with putative nucleolar functions.. Nat Genet.

[52] Walne AJ, Vulliamy T, Marrone A, Beswick R, Kirwan M (2007). Genetic heterogeneity in autosomal recessive dyskeratosis congenita with one subtype due to mutations in the telomerase-associated protein NOP10.. Hum Mol Genet.

[53] Vulliamy T, Beswick R, Kirwan M, Marrone A, Digweed M (2008). Mutations in the telomerase component NHP2 cause the premature ageing syndrome dyskeratosis congenita.. Proc Natl Acad Sci U S A.

[54] Dokal I (2001). Dyskeratosis congenita. A disease of premature ageing.. Lancet.

[55] Yoon A, Peng G, Brandenburger Y, Zollo O, Xu W (2006). Impaired control of IRES-mediated translation in X-linked dyskeratosis congenita.. Science.

[56] Schippert R, Schaeffel F, Feldkaemper MP (2008). Microarray analysis of retinal gene expression in chicks during imposed myopic defocus.. Mol Vis.

[57] Amsterdam A, Sadler KC, Lai K, Farrington S, Bronson RT (2004). Many ribosomal protein genes are cancer genes in zebrafish.. PLoS Biol.

[58] Ender C, Krek A, Friedlander MR, Beitzinger M, Weinmann L (2008). A human snoRNA with microRNA-like functions.. Mol Cell.

[59] Gee HE, Buffa FM, Camps C, Ramachandran A, Leek R (2011). The small-nucleolar RNAs commonly used for microRNA normalisation correlate with tumour pathology and prognosis.. Br J Cancer.

[60] Killian A, Le Meur N, Sesboue R, Bourguignon J, Bougeard G (2004). Inactivation of the RRB1-Pescadillo pathway involved in ribosome biogenesis induces chromosomal instability.. Oncogene.

[61] Jiang H, Lin JJ, Tao J, Fisher PB (1997). Suppression of human ribosomal protein L23A expression during cell growth inhibition by interferon-beta.. Oncogene.

