# Supplementary material for: GRIM-1, a Novel Growth Suppressor, Inhibits rRNA Maturation by Suppressing Small Nucleolar RNAs
Source: PLoS One. 2011 Sep 8;6(9):e24082. doi: 10.1371/journal.pone.0024082 (PMC3169572; doi:10.1371/journal.pone.0024082)
Supplement: Table S1 — Primers used in this study for RT-PCR, cloning and rRNA processing. (DOCX) [file pone.0024082.s001.docx]

**Table S1: Primers used in this study for RT-PCR, cloning and rRNA processing.**

| **ID** | **Sequence (5’ → 3’)** | **Remarks** |
| --- | --- | --- |
| U93-Fwd | CAGACTTGCAGAAAAAGC | SCARNA-13 |
| U93-Rev | CAGTACTTAGTGTTCAACAG |  |
| U92-Fwd | TGGGAGGCTGATACACAAATTGG | SCARNA-8 |
| U92-Rev | ATCTGTCTGCCCCGTATCTG |  |
| U17-Fwd | ATACACCCGGGAGGTCACTC | SNORNA-73 |
| U17-Rev | TGTTTCCTGCATGGTTTGTC |  |
| U19-Fwd | GCTATCCAGGCTCATGTGG | SNORNA-74 |
| U19-Rev | TGTTTGCACCCAGACTAGG |  |
| A50-Fwd | AAGCACTGCCTTTGAACCTG | SNORNA-50 |
| A50-Rev | GAGCTGAAGAGCCCCAGTTA |  |
| hTR-Fwd | AGGCCTGAGTGAGTGTTTGG | TERC |
| hTR-Rev | TCCTATGTGGGGAGTGGAAG |  |
| U3-Fwd | CGTGTAGAGCACCGAAAACC | SNORD-3 (U3) |
| U3-Rev | CACTCAGACCGCGTTCTCTC |  |
| U13-Fwd | CTTTTGTAGTTCATGAGCGTG | SNORD-13 (U13) |
| U13-Rev | GGTCAGACGGGTAATGTGC |  |
| U2-Fwd | ctcggccttttggctaagat | SNRNA-U2 |
| U2-Rev | cgttcctggaggtactgcaa |  |
| U6-Fwd | CGCTTCGGCAGCACATATAC | SNRNA-U6 |
| U6-Rev | AAAATATGGAACGCTTCACG |  |
| 1aF | ccGAATTCgccaccATGCTGACCCCGGCGTTCGAC | Primers for RNA*i*-resistant version of *GRIM-1^*^* |
| 1bF | ccGAATTCgccaccATGTTAACTGCTCTTCTGGCACC |  |
| 1vF | GACACCATGCGAGGAAGTCAGTGAGAGTGCTTTG |  |
| 1vR | CAAAGCACTCTCACTGACTTCCTCGCATGGTGTCTGC |  |
| 1cR | attGGTACCATTATTTGGTGTCTGACAGCCGTC |  |
| Shq1-Fwd | cgcGAATTCgccaccatgataacaccaagattctc | Primers for yeast *shq1* |
| Shq1-Rev | ataggtaccTCATTGTGGGTTCTGCTGCTGCTC |  |
| 1^$^ | gcgggacactcagctaagagc | Used in 1^st^ strand synthesis |
| 2^$^ | ctcgccgcgctctaccttacctacctgg | For unprocessed 18S rRNA using a specific sense primer |
| 3^$^ | gcgcccgtcggcatgtattagctc |  |
| 4^$^ | ggggggcgggtggttggggcgtcc | For unprocessed 18S rRNA using an anti-sense primer |
| 5^$^ | ggcaggatcaaccaggtaggtaagg |  |
| 6^$^ | ggccctgtaattggaatgag | Detects both processed and unprocessed 18S rRNA |
| A^$^ | CTTACGGTACTTGTTGACTATCGGTCTCG | Used in 1^st^ strand synthesis |
| B^$^ | CCCGTCCCCCTCCGAGACGCGACC | For unprocessed 28S rRNA using a specific sense primer |
| C^$^ | CGCTGGGCTCTTCCCTGTTCACTCG |  |
| D^$^ | CTCTCTCCCGTCGCCTCTCCCC | For unprocessed 28S rRNA using an anti-sense primer |
| E^$^ | CGTCTGATCTGAGGTCGCGTCTCGG |  |
| F^$^ | CCAAGTCCTTCTGATCGAGGCCC | Detects both processed and unprocessed 28S rRNA |

^*^ See Figure 4 for comparison with wild-type *GRIM-1* sequence.

^$^ See Figure 5 for relative positions of rRNA primers.

Nucleotides shown in upper-case are regions present in respective genes; small upper-case is the Kozak sequence and upper-case with underline is the enzyme site for cloning.
