# Supplementary material for: GRIM-1, a Novel Growth Suppressor, Inhibits rRNA Maturation by Suppressing Small Nucleolar RNAs
Source: PLoS One. 2011 Sep 8;6(9):e24082. doi: 10.1371/journal.pone.0024082 (PMC3169572; doi:10.1371/journal.pone.0024082)
Supplement: Table S2 — GRIM-1 primers for amplifying exons and sequencing. (DOCX) [file pone.0024082.s002.docx]

**Table S2: GRIM-1 primers for amplifying exons and sequencing.**

| **Primers** | **Fwd primer** | **Rev primer** | **Product** |
| --- | --- | --- | --- |
| **Exon 1** | GGGGAGTTGGAGAGTTTGCG | CGAGCACTGCTCTCTCGACC | 311 bp |
| **Exon 2** | gattgatatattttgatgg | GACATCAAAAGGTCCCCATGG | 260 bp |
| **Exon 3** | cctgcctcagactgccagag | TCACTATATCCCATTTCCCC | 309 bp |
| **Exon 4** | cctatactttttcttcccctag | CTACCAAGTGTAAGCATAACTG | 309 bp |
| **Exon 5** | ggttcttcctgtgctttggc | GAAATGCACATCCCTAGCAGAG | 304 bp |
| **Exon 6** | ctgtatttctagtacaaatatgag | GGCAGTAAACGTGGTACACC | 290 bp |
| **Exon 7** | gacaaatcttccattgggag | CTCTAGACAAGAAAGACTGATGC | 308 bp |
| **Exon 8** | cagtgatcctcagacactgg | GGAATTGCTTGAATTACCCC | 246 bp |
| **Exon 9** | gcagcttttaaatgagaaaagc | TCATTATTATATCTTCACAGAC | 296 bp |
| **Exon 10** | gagctgctttagatatattacc | GGGTAAATAGGTATTTCAGC | 268 bp |
| **Exon 11** | gtataacctgttatccccaggcc | GGTTGAATCCACAGATGTGG | 766 bp |
