# Supplementary material for: GRIM-1, a Novel Growth Suppressor, Inhibits rRNA Maturation by Suppressing Small Nucleolar RNAs
Source: PLoS One. 2011 Sep 8;6(9):e24082. doi: 10.1371/journal.pone.0024082 (PMC3169572; doi:10.1371/journal.pone.0024082)
Supplement: Table S3 — GRIM-1 primers for analyzing mRNA regions present in the preparation. (DOCX) [file pone.0024082.s003.docx]

**Table S3: GRIM-1 primers for analyzing mRNA regions present in the preparation.**

| Primers | Fwd primer | Rev primer | Remarks |
| --- | --- | --- | --- |
| GRIM-1α | GTGGGGAGTTGGAGAGTTTG | CAGACCCCTCGAAGTAGACG | Intactness of GRIM-1α coding region. |
| GRIM-1β | CGTCTACTTCGAGGGGTCTG | TGGTGCCAGAAGAGCAGTTA | Intactness of GRIM-1β coding region. |
| GRIM-1γ | TGCAACGGTTACAGGATGAA | CTTTTCCAAAAAGGCCATCA | Intactness of GRIM-1γ coding region. |

The Ct values obtained using these primers on *GRIM-1* cDNA standard are normalized to unity.

A Relative drift in Ct values for a constant input (unknown or test) are scored as copy number alterations that is a representation of aberrant RNA (gain / loss) in *GRIM-1* mRNA pool.
